# SARS-CoV-2 Vaccination Among Incarcerated People: A Barrier to Overcome

**DOI:** 10.3389/fpubh.2021.704520

**Published:** 2021-08-12

**Authors:** Vito Fiore, Andrea De Vito, Giordano Madeddu, Sergio Babudieri

**Affiliations:** ^1^Unit of Infectious Diseases, Department of Medical, Surgical and Experimental Sciences, University of Sassari, Sassari, Italy; ^2^Italian Society of Penitentiary Healthcare, Sassari, Italy; ^3^SIMSPe School, Viterbo, Italy

**Keywords:** COVID-19, prison settings, vaccination, prevention and control, pandemic

## Introduction

By the end of December 2019, a new severe respiratory syndrome was identified in Wuhan, China (1). In a couple of months, the SARS-CoV-2 disease (COVID-19) was declared as a public health emergency by the WHO (2).

COVID-19 has a broad spectrum of features, such as fever, cough, and dyspnea, while less common symptoms are fatigue, headache, anosmia, ageusia, skin rash, and gastrointestinal symptoms (3–5). A higher risk of disseminated intravascular coagulation and venous thromboembolism has also been described (6). Regarding severity and mortality, different factors have been studied, such as old age (age >65 years), as well as people with preexisting cardiological or cerebrovascular diseases (7–9).

The global health emergency caused by SARS-CoV-2 infection appears unstoppable all over the world, showing a wide spectrum of symptoms, with millions of people being infected and with a very high and unpredictable number of deaths. The spread of the coronavirus certainly does not stop in front of the prison walls and this setting appears at high risk for SARS-CoV-2 infections, given the restricted condition and overcrowding.

Furthermore, incarcerated people are highly vulnerable to coronavirus infection due to their high rates of acute and chronic health disorders, as well as the possibility of less environmental hygiene (10, 11). This makes this environment one of the most important challenges for infection control. More tailored interventions are needed to avoid the spread of SARS-CoV-2 in penitentiary institutes.

## Italian Prison Settings and Preventive Measures During The Pandemic

In 2020, a total of 96,049 people passed through the 190 prisons in the Italian Penitentiary System, with daily attendances of about 52,000−54,000. Over the last year, the number of new incarcerations reduced by 10,921 (9.3%) compared with 2019, and even if it decreased from above 22% to a current 6%, the problem of overcrowding of the system still remains (12).

From the start of the pandemic implementing prevention and control strategies, such as reducing prison permissions, visitors, and giving access exclusively to the essential staff, were attempted (13). Moreover, triage areas, isolation in dedicated prison arms, and collection of biological samples were set-up (13, 14).

## SARS-CoV-2 Spread in Prison Settings

Prevention strategies are fundamental for reducing viral spread, and only some focuses of SARS-CoV-2 have been reported in Italian prisons by literature (11, 15, 16). However, many applied measures resulted in a paradox, reducing incarcerated rights of the people when protecting public health (16). Besides, this is not sufficient for total control and prevention. As a demonstration, a very recent focus in Rome was reported, and the only new measure considered was to evacuate the prison (17).

As per current Ministry of Justice data, SARS-CoV-2 prevalence in Italian prison settings is 0.3% (180/52,517) among incarcerated people and 0.4% (160/36,939) among the prison police. Overall, 3% is actually hospitalized (12).

There is a lack of international literature on SARS-CoV-2 among incarcerated people, and with different screening approaches. Cerrato et al. reported a prevalence of SARS-CoV-2 up to 12.8% in a cohort of 453 incarcerated people (18). Saloner et al. analyzed data on more than 1,000,000 people in federal and state prisons, finding a prevalence of 3.3% (19). de Matos reported 531 cases in Brazilian prison system, with 4.1% of deaths (20). Njuguna et al. reported results from a serial laboratory testing in Luisiana during 2020. In this case, out of 98 people tested for SARS-CoV-2, 72% were positive (21). Marco et al. reported data on a SARS-CoV-2 outbreak in a Barcelona prison. Among 148 incarcerated people who underwent nasopharyngeal swab, 22.3% tested positive for SARS-CoV-2 (22). The available study findings have been reported in Table 1.

**Table 1:** Studies on SARS-CoV-2 spread in prison settings revised in our literature search (values calculated from available data).

**Literature**	**Country**	**Tested population, *n***	**SARS-CoV-2 infected, *n* (%)**	**Hospitalized, *n* (%)**	**Deaths *n* (%)**
Cerrato et al. (18)	Italy	453	58 (12.8%)	5 (8.6%)	3 (5.2%)
de Matos (20)	Brazil	NA	531 (NA)	NA	22 (4.1%)
Marco et al. (22)	Spain	148	33 (22.3%)	1 (3%)	–
Njuguna et al. (21)	U.S.	98	71 (72%)	NA	NA
Saloner et al. (19)	U.S.	1,295,285	42,107 (3.3%)	NA	510 (1.2%)

Considering the available literature, the reported infection prevalence is highly variable, probably also for the different testing approaches among prison population (e.g., symptoms based or extended testing programs). Substantially, these data highlighted the need for more tailored interventions and a homogenous approach in prison settings, with better coordination from the health authorities.

## Facing SARS-COV-2 Spread in Prison Settings: Perspectives and Concerns

When considering international reports, correctional institutes were involved in multiple SARS-CoV-2 outbreaks worldwide, with crude COVID-19 death rate higher than the outside community (19).

As well it is known, correctional settings are at higher risk for SARS-CoV-2 spread due to overcrowding, poor air exchange, high population turnover, and less healthcare provision. For this reason, experts continue to point out the need to include prisons as prioritized environments regarding SARS-CoV-2 vaccine. Despite this, incarcerated people have not been included as vaccine trial populations (23). Many trials have been conducted to evaluate the efficacy and safety of the different proposed vaccines, but none enrolled people living in correctional facilities (24).

## Discussion

Social problems have a high impact among incarcerated people. Disparities on ethnicity, gender, age, and class have been well-described in literature (25). Furthermore, a high prevalence of people who inject drugs is present in Italian penitentiary settings (12). As a consequence, reaching out to patients for the vaccine administration after coming back to freedom would certainly be more difficult than during their stay in the closed prison community. Numerous papers have been published, demonstrating how incarceration is sometimes an extraordinary occasion for infectious diseases prevention and control (26–28). Also for these reasons, the vaccination of people living in a prison could also protect the entire community. Reinhart and Chen demonstrated how almost 16% of SARS-CoV-2 infected people in Chicago were correlated with the Cook County Jail outbreak (29). An outbreak inside prisons does not concern only the incarcerated people but also all people working in prison, in courts, and their relatives. In this regard, it is necessary to remember that, after having limited or closed access to relatives of detainees, the only vehicle that SARS-CoV-2 has to enter the closed prison communities is those who work there. For this reason, vaccination campaigns should place all prison workers such as policemen, administrators, educators, sociologists, and health professionals in the first place to prevent the virus from attacking the prison population. Incarcerated people should be vaccinated immediately after for the numerous reasons already outlined. If the vaccination campaign would be implemented, it could be considered to perform serology and nasopharyngeal swab only to new entries, both incarcerated people and operators. In this way, the virus would be locked out from the closed community. This kind of approach could be applied in all penitentiary settings, particularly thanks to lower costs of rapid testing.

## Conclusions

Correctional facilities have all the conditions to be considered as an integral part of the global community and to not be considered as separate entities.

In such a restricted environment, not having the free choice of being able to join a vaccination program does not result in protection, but into a further, not imposed by the Judge, restriction on individual freedom, together with the real risk of a coronavirus infection without having the possibility of self-determination in the choice of treatments as one has in freedom.

Prioritization for SARS-CoV-2 vaccine distribution among incarcerated people is still a challenge. Actually, the efficacy and safety of all the vaccines have been well-demonstrated. However, distribution in correctional settings remains a barrier that has to be overcome as soon as possible.

## Author Contributions

All authors contributed to manuscript conceiving, drafting, and approved the final version.

## Conflict of Interest

The authors declare that the research was conducted in the absence of any commercial or financial relationships that could be construed as a potential conflict of interest.

## Publisher's Note

All claims expressed in this article are solely those of the authors and do not necessarily represent those of their affiliated organizations, or those of the publisher, the editors and the reviewers. Any product that may be evaluated in this article, or claim that may be made by its manufacturer, is not guaranteed or endorsed by the publisher.
